# Gas and Liquid Phase Imaging of Foam Flow Using Pure Phase Encode Magnetic Resonance Imaging

**DOI:** 10.3390/molecules26010028

**Published:** 2020-12-23

**Authors:** Alexander Adair, Sebastian Richard, Benedict Newling

**Affiliations:** MRI Centre, University of New Brunswick, 8 Bailey Dr., Fredericton, NB E3B5A3, Canada; srichar6@unb.ca (S.R.); bnewling@unb.ca (B.N.)

**Keywords:** foam flow, magnetic resonance imaging, velocity mapping, pipe flow, two-phase flow

## Abstract

Magnetic resonance imaging (MRI) is a non-invasive and non-optical measurement technique, which makes it a promising method for studying delicate and opaque samples, such as foam. Another key benefit of MRI is its sensitivity to different nuclei in a sample. The research presented in this article focuses on the use of MRI to measure density and velocity of foam as it passes through a pipe constriction. The foam was created by bubbling fluorinated gas through an aqueous solution. This allowed for the liquid and gas phases to be measured separately by probing the ^1^H and ^19^F behavior of the same foam. Density images and velocity maps of the gas and liquid phases of foam flowing through a pipe constriction are presented. In addition, results of computational fluid dynamics simulations of foam flow in the pipe constriction are compared with experimental results.

## 1. Introduction

Foams are integral to many industrial and consumer applications, including petroleum (e.g., enhanced oil recovery), cosmetics (e.g., shaving cream), and food science (e.g., beer foam). Foams have been studied extensively, with investigations commonly focusing on liquid holdup and drainage [1,2,3,4,5,6], and bubble size distribution [7,8]. Foam measurements have been performed using a variety of methods, both invasive and non-invasive, such as optical measurements with insertion probes [8], gamma ray absorption [9], and tracer particles [10]. Foam has also been studied using magnetic resonance imaging (MRI) with particular focus on foam drainage [1,2,3,4] and bubble size distribution [7]. At its core, MRI reports the position of nuclei in a sample based on their position within a magnetic field gradient. This information on the density and position of nuclei is used to create images of the sample. Data from an MRI experiment can also be sensitized to provide additional information on sample behavior. Of particular interest for the experiments described in this paper is sensitizing MRI to the motion of nuclei in a sample, allowing for velocity mapping.

MRI has several benefits for the study of foams when contrasted with other flow measurement techniques. MRI is non-optical; therefore, the internal behavior of a foam can be studied even in visually opaque foams. MRI is also non-invasive; therefore, it does not impede movement or otherwise interfere with the structure of the foam. This is especially important in the case of delicate foams which might break down under the scrutiny of an invasive measurement technique. MRI is also capable of imaging in one to three spatial dimensions. In addition, MRI measurements can be sensitized to different nuclei. This is of particular importance because separate measurements of the liquid and gas phases of a foam can be performed by choosing the liquid and gas components of the foam judiciously.

When considering measurements of foam, an important consideration is the effect that magnetic susceptibility differences between the gas and liquid phases have on results. These magnetic susceptibility differences manifest as image artifacts in typical frequency-encoding techniques. On the other hand, a pure phase encoding technique provides images free of artifacts due to magnetic susceptibility differences at the gas–liquid interfaces in foams. Of particular interest for the study of foams are MRI measurement techniques that are well-suited for measuring signals with short lifetimes. This area of study is well developed with both frequency encoding techniques (such as UTE (ultra-short echo time imaging) and ZTE (zero echo time imaging)) and phase encoding techniques (such as SPI (single point imaging) and SPRITE (Single Point Ramped Imaging with T_1_-Enhancement)) [11]. While phase encoding measurement techniques like SPRITE have the benefit of reduced image artifacts due to magnetic susceptibility differences, they are typically much slower measurements compared to frequency encoding techniques like UTE and ZTE.

The SPRITE [12] measurement technique is a pure phase encoding technique and is well-suited for creating density images of foam. To study the movement of foam, data acquired during a SPRITE measurement can be sensitized to motion with the addition of pulsed field gradients (PFGs) [13]. Motion sensitization was incorporated into the measurements used in these experiments by preceding the typical SPRITE imaging with a PFG preparation phase. In particular for the study of foam flow, the alternating pulsed gradient stimulated echo (APGSTE) preparation [14] was used because it reduces the effects of the background magnetic field gradients on the data. The benefits of the APGSTE preparation are applicable to the foam flow studied for this paper, but they would be increasingly important in applied foam flows with extreme magnetic susceptibility-induced magnetic field gradients (such as enhanced oil recovery). Motion-sensitized measurements using the APGSTE preparation and SPRITE imaging have previously been used to study other two-phase flow systems and flow in porous media [15,16].

This paper reports the first use of APGSTE-SPRITE to study foam flow. In addition, this paper also reports the first use of APGSTE-SPRITE to create velocity maps of both phases of a two-phase flow. This was achieved by bubbling a fluorinated gas through a water solution to create the foam. Information on the liquid phase was acquired by measuring signals from ^1^H nuclei, and information on the gas phase was acquired by measuring signals from ^19^F nuclei. The experimental apparatus was designed such that gas and liquid phase measurements were taken sequentially, without interrupting the foam flow. This paper demonstrates the use of motion-sensitized SPRITE MRI as a measurement technique for the study of foam flow and the comparison between gas and liquid phase velocity maps. Further, this paper presents the use of the Herschel–Bulkley viscosity model as a means of modelling the foam flow through the pipe, rather than more computationally intensive methods [17,18]. Computational fluid dynamics (CFD) simulation results created using this model are presented and found to be only superficially similar to experimental results, suggesting the future use of MRI measurements to guide CFD simulations of foam behavior.

## 2. Results

**Figure 1:**
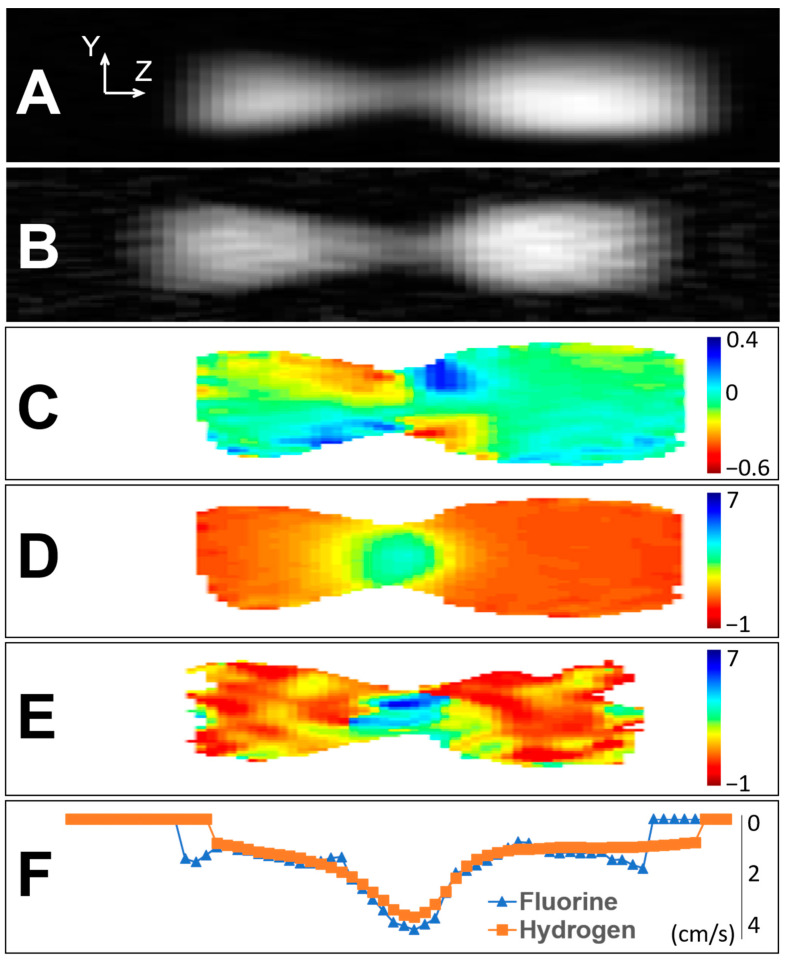
Density and velocity maps of the foam flow for the liquid phase (hydrogen signal) and the gas phase (fluorine signal). (**A**) Density image of the liquid phase. (**B**) Density image of the gas phase. (**C**) Y-component of velocity map of the liquid phase. (**D**) Z-component velocity map of the liquid phase. (**E**) Z-component velocity map of the gas phase. **(F**) Z-component of velocity for both phases, averaged across the width of the pipe. All speeds are in units of cm/s, and bulk flow is from left to right. Uncertainty in speed values was estimated from variations in speed in a region downstream from the constriction throat where speed should be uniform. Uncertainty in speed for (**D**) is ± 0.2 cm/s and for (**E**) is ± 1.7 cm/s.

The density and velocity maps of the flowing foam for hydrogen and fluorine signal are shown together in Figure 1. As mentioned previously, hydrogen signal reports on the liquid phase of the foam, while fluorine signal reports on the gas phase. Figure 1A,B are 2D images of the foam for hydrogen and fluorine signal, respectively. Since the images are time-averaged and two-dimensional projections, the images do not show individual bubbles in the foam, but rather, signal intensity is proportional to the averaged density (of hydrogen or fluorine) in each image. The effect of buoyancy is evident in the hydrogen density image—there is a gradation of density in the Y-direction (i.e., vertically in the lab frame) with higher density shown at the bottom of the pipe in the hydrogen image, indicating a separation of the two foam phases. Buoyancy is less obvious in the fluorine density image, likely due to a poorer signal-to-noise ratio than in the hydrogen image.

Figure 1C is the map of the Y-component of velocity for the hydrogen signal, and it shows expected results. The foam moved in towards the pipe axis as it flowed into the constriction and the pipe diameter decreased. The foam moved away from the pipe axis as the pipe diameter increased past the narrowest point. Figure 1D is the map of the Z-component of velocity (i.e., along the pipe axis) for the hydrogen signal. The results were as expected. The flow speed along the pipe axis increased as the diameter of the pipe decreased in the constriction. The flow speed decreased as the diameter increased past the narrowest point, and a jet was present. Figure 1E is the map of the Z-component of velocity for the fluorine signal. The expected behavior of higher speeds being present at the narrowest point in the pipe was apparent, although the results were less clear than the hydrogen velocity map due to the poor signal-to-noise ratio (SNR) of the fluorine signal. Figure 1F shows velocity profiles of the Z-component for hydrogen and fluorine signal taken along the pipe axis, averaged across the width of the pipe. Although the uncertainty in velocity values for the fluorine signal was high due to the low SNR, the comparison of centerline profiles indicated that the velocities along the pipe axis were comparable with hydrogen.

**Figure 2:**
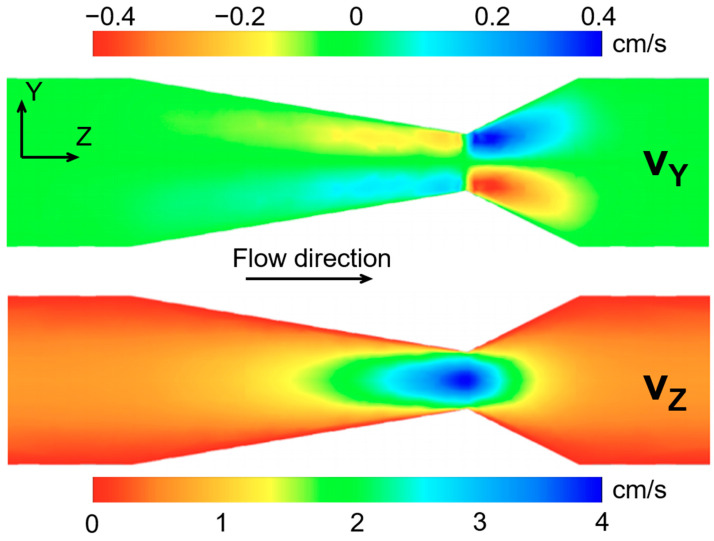
Simulation results of foam flow through the pipe constriction using a Herschel–Bulkley fluid viscosity model, a foam quality of 0.75, and a flow rate of 0.0009 L/s. The bulk flow direction is from left to right. The figure on top shows the results for the Y-component of velocity, and the figure on bottom shows the results for the Z-component of velocity.

Figure 2 shows the CFD simulation results of the foam flow through the pipe constriction, for which representative parameter values were taken from the literature (see Materials and Methods). The simulation results superficially resembled the experimental results but were not identical, which encourages future use of the MRI results to guide the development of more accurate simulations. The figure at the top shows the Y-component of velocity. The general behavior was similar to the results shown in Figure 1C (the Y-component velocity map of the liquid phase from experimental data). The fluid moved towards the pipe axis as the pipe diameter decreased, and it moved away from the axis as the diameter increased past the narrowest point. The figure at the bottom of Figure 2 shows the Z-component of velocity. The general behavior was similar to that of Figure 1D (the Z-component velocity map of the liquid phase, from experimental data). The flow speed attained its highest value at the narrowest point in the constriction, and the jet is present in both images.

## 3. Discussion

Magnetic resonance imaging has several advantages over other flow measurement techniques that make it well-suited for studying foam flow. It is able to map directly sample density to position (to create density images as shown in Figure 1) and is able to create density images for separate nuclei present in the sample. The hydrogen and fluorine density maps created of the foam flow demonstrate the effectiveness of MRI at studying the two phases (gas and liquid) of the foam. Buoyancy is clearly evidenced by the gradation in density, demonstrating the effect of gravity on the foam for both phases.

The APGSTE-SPRITE preparation-readout technique incorporated motion information into the images, and velocity maps of the foam flow were created. The velocity maps do not provide a snapshot image for the flow but are instead time-averaged over several hours as a consequence of signal averaging to improve SNR. The velocity maps show expected flow behavior and demonstrate that APGSTE-SPRITE is well-suited for imaging velocity. Primarily, when comparing the Z-component of velocity for the hydrogen and fluorine signals (i.e., liquid and gas phases), the velocity values are comparable, within uncertainty. In addition, CFD simulation results show only similar behavior to the foam flow when compared with experimental results. This gives incentive to continue the development of the Herschel–Bulkley viscosity model for simulating foam flow in a pipe constriction. Typical values were taken from the literature to create the initial simulations, but future work will continue improving the simulations of the foam flow by analyzing a wider range of values guided by the MRI results.

While the APGSTE-SPRITE technique has been demonstrated in this paper for the study of foam flow in a pipe constriction, it can easily be adapted to studying flow systems where the effects of magnetic susceptibility effects are more extreme, such as in enhanced oil recovery or froth flotation. Additionally, a principal focus of this work was the adaptation of the measurement technique to multi-nuclear study of flow. In future work, a multi-nuclear measurement approach could be used to study flow in rock cores [16] and bubbly flow in pipes [19]. The APGSTE-SPRITE measurement could also be used for studies in a variety of other flow systems, such as rock fractures [20] and polymer flooding [21].

## 4. Materials and Methods 

Measurements were performed using a 2.4 T horizontal bore superconducting magnet (Nalorac, CA, USA) with homebuilt magnetic field gradient hardware capable of delivering maximum gradient amplitudes of 0.26 T/m in the Z-direction (oriented along the magnet bore) and 0.28 T/m in the Y-direction (oriented vertically). Radio frequency excitation and signal detection was accomplished using two homebuilt birdcage coils (RF probes) driven by a 2 kW Tomco amplifier (Tomco, Australia). The RF probes were designed and tuned such that one measured the signal from hydrogen (^1^H) in the sample and the other measured the signal from fluorine (^19^F).

**Figure 3:**
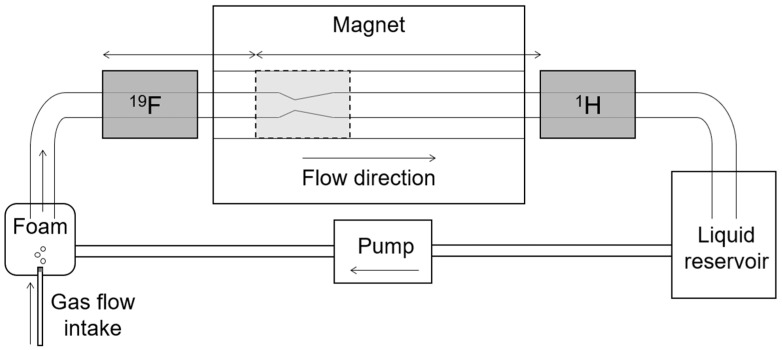
Schematic of the flow apparatus. Foam was created by bubbling C_4_F_8_ gas through a liquid solution and flowed through a pipe placed in the magnet. A constriction in the pipe was the region of interest. Fluorine signal (gas phase) was measured by inserting the ^19^F RF probe and removing the ^1^H RF probe. Hydrogen signal (liquid phase) was measured by inserting the ^1^H RF probe and removing the ^19^F probe. The shuttling of probes was accomplished without interrupting the foam flow.

A plexiglass pipe (I.D. = 1.9 cm) was placed inside the superconducting magnet with the pipe axis oriented in the Z-direction. A constriction in the pipe with a minimum inner diameter of 0.6 cm (see Figure 3) was positioned inside the imaging region of the apparatus. Foam was generated with an aqueous solution of distilled water, sodium dodecyl sulfate (SDS) (1.5 g/L), and glycerol (30 mL/L). The aqueous solution was kept in a reservoir, and a peristaltic pump (Masterflex, Cole-Parmer Montreal, Canada) moved the solution from the main reservoir to a smaller reservoir used to create the foam. The foam was created by flowing octafluorocyclobutane (C_4_F_8_) gas through a sparger (50 μm pore size) immersed in the aqueous solution. The flow rate of the peristaltic pump was set to maintain a constant liquid level in the smaller reservoir. The foam flowed through the plexiglass pipe inside the magnet before returning to the reservoir containing the foaming solution.

The flow rate of the C_4_F_8_ gas was set at the beginning of the experiment and maintained at that flow rate to ensure a consistent foam for all measurements. The flow rate was chosen to fit with experimental constraints imposed by the imaging field of view (15 cm in the Z-direction) and achievable magnetic field gradient amplitudes. A conventional RF probe design would have required stopping the foam flow and disassembling the flow network in order to switch between hydrogen and fluorine measurements. The RF probes were modified to allow them to be moved in and out of the region of interest without interrupting the foam flow, thus allowing for separate gas and liquid phase measurements of a continuously flowing foam (see Figure 3). The probe switching was implemented in the following manner: the flow apparatus was set up with a flowing foam and the ^19^F RF probe in the imaging position. All measurements were acquired of the ^19^F signal. Without interrupting the foam flow, the ^19^F RF probe was manually removed from the magnet by sliding it along the pipe. The ^1^H RF probe was then manually inserted from the opposite side of the magnet by sliding it along the pipe and into the imaging region. All measurements were then acquired of the ^1^H signal. It took several minutes to switch the RF probes and verify their position. The process could be automated, but it was not in this proof-of-principle measurement. The foam solution was doped with gadolinium chloride (GdCl_3_) to attain a spin-lattice relaxation time constant from the hydrogen signal of T_1_ = 327 ms. The T_1_-relaxation time constant of the C_4_F_8_ gas was T_1_ = 56 ms. Both values were measured in a stationary foam using standard inversion recovery methods. The T_2_* relaxation time constant of the foam from the hydrogen signal was T_2_* = 0.4 ms, which is indicative of considerable susceptibility-induced line-broadening (typical T_2_* in homogeneous liquids being 3–5 ms in the same magnet).

Hydrogen and fluorine density images of the foam flow were acquired using the 2D Spiral SPRITE MRI measurement technique [22]. Interleaved k-space measurement trajectories were used for this experiment such that each individual trajectory had a short duration. The duration of the measurement trajectories was set to be less than the T_1_-relaxation time constants (see above) in order to ensure that signal sensitization from the preparation phase persisted through all acquired imaging data points. The ^1^H RF probe was used to create density images of the liquid phase of the foam. The hydrogen density image was created from 64 scans, with a total imaging time of 16 min. The ^19^F RF probe was used to create density images of the gas phase of the foam. The fluorine density image was created from 1024 scans, with a total imaging time of 65 min. All images created from these measurements are time-averaged and do not show an instantaneous image of the foam. The field of view for both density images was 15 cm in the Z-direction (oriented along the pipe axis) and 3 cm in the Y-direction (oriented vertically), with a nominal resolution of approximately 2 mm/pixel in the Z-direction and 0.5 mm/pixel in the Y-direction. The phase-encoding time (the time interval between sample excitation and signal detection) was t_p_ = 150 μs. Flow-induced smearing effects caused by sample movement during the phase-encoding interval were not present due to the short phase-encoding time and slow flow speeds.

**Figure 4:**
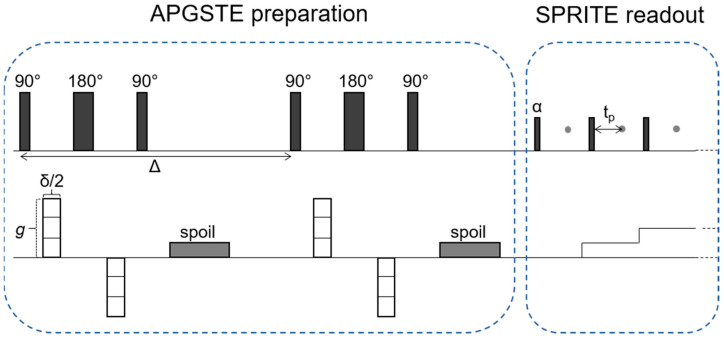
A schematic of the APGSTE-SPRITE pulse sequence [15]. Each individual SPRITE k-space measurement trajectory is preceded by an APGSTE preparation to ensure all measured data are motion sensitized. The parameters shown are as follows: *g* is the amplitude of the motion-sensitizing PFG, *δ*/2 is the duration of the PFG, Δ is the flow evolution time, *α* is the flip angle of the imaging RF pulses in the SPRITE readout, and *t_p_* is the phase encoding time (the time between sample excitation and signal detection).

Velocity maps of the flowing foam were created using a preparation readout approach. Velocity information was introduced into the measured signal during the preparation stage with an alternating pulsed gradient stimulated echo (APGSTE) pulse sequence, which encoded signal phase based on the distance traveled by the sample during a set time interval. A Spiral SPRITE readout followed the preparation, which imposed spatial information onto the prepared sample magnetization. A Fourier transformation of the acquired data was used to construct an image of the sample. To ensure that motion sensitization information was contained in all acquired data, each individual, short-duration measurement trajectory of the Spiral SPRITE imaging readout was preceded by an APGSTE preparation, and sample magnetization was allowed to fully recover after the previous imaging readout and before the next preparation was applied. A schematic of the APGSTE-SPRITE measurement pulse sequence is shown in Figure 4.

The APGSTE-SPRITE measurement was repeated three times with varying amplitude of the motion-sensitizing gradient pulses (*g* in Figure 4). Acquiring measurements with different values of *g* builds up information in a reciprocal displacement space (or *q*-space) where *q* is defined as $q=\gamma \delta g{\left(2\pi \right)}^{-1}$, where γ is the gyromagnetic ratio of the nucleus. A mean velocity value *v_avg_* was extracted for each pixel in the image by linear fitting the signal phase against *q* across the three measurements according to Equation (1):

(1) $$v_{avg}=\frac{1}{2\pi }\frac{d}{dq}\left(\tan^{-1}\frac{Im\left(S\right)}{Re\left(S\right)}\right)$$

where *Im*(*S*) and *Re*(*S*) are the imaginary and real components of the measured signal, respectively [16].

Velocity maps were created in the Y- and Z-directions for the liquid phase of the foam (hydrogen signal) and in the Z-direction for the gas phase of the foam (fluorine signal). A velocity map of the gas phase in the Y-direction was not acquired due to the large-amplitude, motion-sensitizing gradients that would have been required in that case. The duration of the motion-sensitizing pulses and flow evolution time are *δ* = 0.6 ms and Δ = 6.15 ms, respectively. For the hydrogen measurements, the maximum amplitude of the motion-sensitizing gradients was 0.037 T/m in the Z-direction and 0.079 T/m in the Y-direction. For the fluorine measurements, the maximum amplitude of the motion-sensitizing gradients was 0.039 T/m in the Z-direction.

Computational fluid dynamics simulations of the foam flow were performed using the SimScale computer-aided engineering software (www.simscale.com) [23] with the OpenFOAM CFD modelling module (www.openfoam.com) [24,25]. The foam was modelled as a Herschel–Bulkley fluid, which is a non-Newtonian, shear thinning fluid. This model has previously been used to describe the flow of aqueous foams. A foam quality value (defined as the ratio of the volume of the gas in the foam to the total liquid and gas volume of the foam) of 0.75 was used for the simulations, based on parameters provided in [17]. A foam quality factor below 0.52 represents a foam that consists of spherical bubbles that are not in contact with each other. Between 0.52 and 0.96, the bubbles are in contact with each other with a corresponding increase in viscosity. Above 0.96, the foam becomes a mist with a corresponding decrease in viscosity. A range of foam quality values of 0.6 to 0.85 was also simulated, and 0.75 was chosen as representative of this range because it proved possible to mimic the experimental results with this simulation.

## Data Availability

The data presented in this study are available on request from the corresponding author. The data are not publicly available due to non-standard, proprietary formatting, which will necessitate explanation on sharing.
